# The Transcriptional Targets of Mutant FOXL2 in Granulosa Cell Tumours

**DOI:** 10.1371/journal.pone.0046270

**Published:** 2012-09-28

**Authors:** Roseanne Rosario, Hiromitsu Araki, Cristin G. Print, Andrew N. Shelling

**Affiliations:** 1 Department of Obstetrics and Gynaecology, University of Auckland, Auckland, New Zealand; 2 Department of Molecular Medicine and Pathology, University of Auckland, Auckland, New Zealand; 3 Bioinformatics Institute of New Zealand, University of Auckland, Auckland, New Zealand; Institut Jacques Monod, France

## Abstract

**Background:**

Despite their distinct biology, granulosa cell tumours (GCTs) are treated the same as other ovarian tumours. Intriguingly, a recurring somatic mutation in the transcription factor Forkhead Box L2 (*FOXL2*) 402C>G has been found in nearly all GCTs examined. This investigation aims to identify the pathogenicity of mutant *FOXL2* by studying its altered transcriptional targets.

**Methods:**

The expression of mutant *FOXL2* was reduced in the GCT cell line KGN, and wildtype and mutant *FOXL2* were overexpressed in the GCT cell line COV434. Total RNA was hybridised to Affymetrix U133 Plus 2 microarrays. Comparisons were made between the transcriptomes of control cells and cells altered by *FOXL2* knockdown and overexpression, to detect potential transcriptional targets of mutant *FOXL2*.

**Results:**

The overexpression of wildtype and mutant *FOXL2* in COV434, and the silencing of mutant *FOXL2* expression in KGN, has shown that mutant *FOXL2* is able to differentially regulate the expression of many genes, including two well known *FOXL2* targets, *StAR* and *CYP19A*. We have shown that many of the genes regulated by mutant FOXL2 are clustered into functional annotations of cell death, proliferation, and tumourigenesis. Furthermore, *TGF-β* signalling was found to be enriched when using the gene annotation tools GATHER and GeneSetDB. This enrichment was still significant after performing a robust permutation analysis.

**Conclusion:**

Given that many of the transcriptional targets of mutant *FOXL2* are known *TGF-β* signalling genes, we suggest that deregulation of this key antiproliferative pathway is one way mutant *FOXL2* contributes to the pathogenesis of adult-type GCTs. We believe this pathway should be a target for future therapeutic interventions, if outcomes for women with GCTs are to improve.

## Introduction

Granulosa cell tumours of the ovary (GCT) are the predominant type of ovarian sex-cord tumour, yet they comprise approximately 5% of all malignant ovarian neoplasms [1], [2]. Their rarity poses a limitation in our understanding of their aetiology and molecular pathogenesis. Divided into two distinct subtypes: adult and juvenile; the tumour cells of GCTs demonstrate several morphological, biochemical and hormonal features of normal proliferating pre-ovulatory granulosa cells. Women with GCTs tend to present with symptoms of excessive oestrogen secretion by the tumour, enabling the disease to be detected at an early stage due to the consequent symptoms of abnormal uterine bleeding, menorrhagia or cycle disturbances. However, GCTs are characterised by slow growth and a tendency to relapse, requiring patients with GCTs to undergo prolonged follow-up, as recurrences have been known to occur even forty years after the initial diagnosis [3], [4]. The overall relapse rate for women with adult-type GCTs is approximately 30%, however 70–80% of women with recurrent disease will die from GCTs [5].

In contrast to ovarian epithelial tumours, GCTs are a relatively homogenous tumour, likely to have arisen from a limited set of molecular events in specific signalling pathways [6]. Yet current GCT treatment strategies are modelled on the behaviour of ovarian epithelial tumours. Given the numerous differences between granulosa cells, and ovarian surface epithelial cells, it is likely that GCTs may require a specific treatment based on the molecular defects in the tumour itself, rather than being treated like all ovarian tumours, which only share with GCTs a common location, the ovary. With this in mind, a range of genes important in normal granulosa cell biology, as well as their relevant signalling pathways have been investigated as putative candidates involved in GCT pathogenesis (refer to Jamieson & Fuller, 2012 for a comprehensive review).

*FOXL2* belongs to the large family of forkhead FOX transcription factors, and its expression is strongly maintained in granulosa cells throughout life [7], [8]. Furthermore, the overall phenotype of *FOXL2* knockout mice models confirms that this gene is critical for the proper differentiation of granulosa cells [9], [10]. *FOXL2* expression has also been observed in the developing eyelid, thus notably involving this gene in the pathogenesis of blepharophimosis ptosis epicanthus inversus syndrome (BPES), with or without accompanying premature ovarian failure (POF). Considering that POF is part of the phenotypic spectrum of *FOXL2* mutations, *FOXL2* was assumed to be a possible candidate for POF in the absence of BPES. Indeed previous work on this gene has identified two novel *FOXL2* variants in two women with isolated cases of POF from New Zealand and Slovenia [11].

In 2009, the landmark study by Shah et al identified a recurring somatic mutation 402C>C in the gene *FOXL2* [12]. This mutation was confirmed to be present in 97% if adult GCTs subsequently tested, and in much lower proportions (10%) in juvenile GCTs; a finding that has been replicated in independent cohorts of GCTs [13], [14], [15]. Most interestingly, this mutation was not found in any other sex-cord stromal tumours, nor in any unrelated ovarian or breast tumours [16], [17].

The 402C>G mutation results in an amino acid substitution of tryptophan for cysteine (C134W) [12], [13], which is located in the second wing on the surface of the forkhead domain. Computer modelling suggests this alteration does not disrupt the folding of the *FOXL2* forkhead domain or its interactions with DNA. In addition it has been shown that mutation does not affect the localisation of the *FOXL2* protein [18]. Therefore it is speculated that the pathogenicity of mutant *FOXL2* occurs through changes to its interactions with other proteins. Such candidate proteins include the SMAD transcription factors and the effectors of *TGF-β* and *BMP* family signalling [19]. To date, there have been few publications exploring the pathogenicity of mutant *FOXL2*. The transactivation capability of mutant *FOXL2* on known wildtype *FOXL2* targets has been investigated, whilst one report describes the inability of mutant *FOXL2* to elicit an effective apoptotic signalling cascade to be partially accountable for the pathophysiology of GCT development [18], [20]. Lastly, the aromatase gene has been identified as a direct target of mutant *FOXL2* with the use of promoter-luciferase constructs [21]. Given this specific *FOXL2* mutation is found in nearly all adult-type GCTs examined, it is clear this mutation must confer some survival advantage even in the heterozygous state. However further studies are required to understand the specific molecular effects of this compelling *FOXL2* mutation.

The two well characterised human derived GCT lines, KGN and COV434 have each been screened by us and others for the *FOXL2* mutation. The KGN line, established from a 67 year old woman with a recurrent metastatic GCT [22], was shown to be heterozygous for the 402C>G mutation [13]. However the COV434 line was shown to contain wildtype *FOXL2* [13]. COV434 was also derived from a recurrent metastatic GCT, but from a much younger woman aged 27 [23]. For these reasons, KGN is considered to be representative of an adult-type GCT and COV434 a juvenile-type GCT, making both these cell lines useful models when studying the tumour properties of each subtype. Interestingly, the two cell lines also differ in their expression of *FOXL2*. Whilst KGN is shown to have abundant *FOXL2* expression, this gene is almost absent in COV434 [13], [21]. This finding correlates to observations made by Kalfa et al, who noted *FOXL2* immunochemistry to be decreased or absent in juvenile-type GCTs [24], [25].

The aim of this investigation was to identify *FOXL2* transcriptional targets with relevance to GCT by analysing the effect of altering *FOXL2* expression on the transcriptome of GCT cell lines. This was achieved in two ways. Mutant *FOXL2* was targeted for gene silencing in KGN, whereas both wildtype and mutant *FOXL2* were overexpressed in COV434. Total RNA from each perturbation experiment was then hybridised to Affymetrix U133 Plus 2 microarrays. By perturbing the expression levels of wildtype and mutant *FOXL2* in these two cell lines, we wanted to see altered expression in unique suites of genes that reflect the activity of certain molecular pathways.

## Methods

### Cell Lines

Two human derived GCT cell lines KGN and COV434 were obtained from the Riken Cell Bank and the American Tissue Culture Collection (ATCC), respectively. Both cell lines were cultured in RPMI 1640 supplemented with 10% fetal bovine serum (FBS), 1% L-glutamine and 1% non essential amino acids, and maintained at 37°C in 5% CO_2_.

### Plasmid Constructs

The wildtype *FOXL2* plasmid (p*FOXL2*wt) was purchased from OriGene Techonologies (SKU SC126215, Rockville, MD). The 402 C>G mutant *FOXL2* (p*FOXL2*m) construct was produced using site directed mutagenesis on p*FOXL2*wt, performed by Mutagenex http://www.mutagenex.com/01_Mutagenesis/02_Site.html (Hillsborough, NJ). The empty vector control was made by excising the *FOXL2* sequence from p*FOXL2*wt with NotI and ligating the vector backbone. The constructs were confirmed to be correct by DNA sequence analysis.

### Transient Transfections

For siRNA knockdown of mutant *FOXL2*, KGN cells, passage 8, were seeded at a density of 300,000 cells per well of a six well plate. Cells were transfected with *FOXL2* stealth RNAi™ (cat#HSS101080 Invitrogen, NZ) or a GC matched control using Lipofectamine 2000 and Opti-MEM serum free media (Invitrogen, NZ). RNAi-lipid complexes were removed 7 h post transfection and replaced with complete media. For overexpression of wildtype or mutant *FOXL2*, COV434 cells, passage 9, were seeded at a density of 300,000 cells per well of a six well plate. Cells were transfected with 1 µg of control (empty vector), p*FOXL2*wt, or p*FOXL2*m using Lipofectamine 2000 and Opti-MEM serum free media. Plasmid-lipid complexes were removed 7 h post transfection and replaced with complete media. All transfections were performed in triplicate.

### RNA Extraction and cDNA Synthesis

Cells were harvested 24 h post transfection and total RNA was extracted using TRIzol® reagent (Ambion, NZ). Chloroform was added to the TRIzol® to separate the phases, and the aqueous phase was combined with 70% ethanol and passed through an RNeasy column (Qiagen, Australia) according to manufacturer’s instructions. The integrity of the total RNA extracted was verified using the Experion™ automated electrophoresis system (BioRad, NZ). 0.5 µg of total RNA was reverse transcribed to cDNA using oligo(dT) primers and SuperscriptIII reverse transcriptase (Invitrogen, NZ) according to the manufacturer’s instructions. The cDNA synthesis reaction was then diluted with 100 µL of sterile water for RT-qPCR.

### RT-qPCR

RT-qPCR was performed to assess the level of mutant *FOXL2* knockdown in KGN, the level of overexpression of wildtype and mutant *FOXL2* in COV434, and to validate both sets of microarray results. RT-qPCR primers for microarray validation were designed within the probeset region for Affymetrix U133 Plus 2 microarrays using the Primer3 software. Primer pair amplification efficiencies were calculated with the LinReg PCR applet [26]. Each reaction was performed in a final volume of 10 µL, with 1x SYBR Green master mix, 20 pmol of each primer and 2 µL of diluted cDNA. Each cDNA sample was analysed in triplicate. The expression levels of the target gene were normalised to the expression of three most stable housekeeping genes determined with the use of SLqPCR package in R. Data analysis for normalisation, relative quantification of gene expression and calculation of standard deviations was performed as outlined by Vandesompele et al [27].

### Western Blotting

Western blotting was performed to assess the level of overexpression of wildtype and mutant *FOXL2* in COV434. Cells were harvested 24 h post transfection in RIPA buffer and lysates were incubated with Benzonase® nuclease (Novagen, CA) to remove any residual DNA/RNA. Protein separation was performed on Mini protean TGX precast gels (BioRad, NZ) with Laemmli running buffer. Proteins were transferred onto polyvinylidene fluoride membranes. Western blotting was conducted with an anti-*FOXL2* N-terminus polyclonal antibody [7] at a dilution of 1∶500 overnight at 4°C, or a monoclonal anti-vimentin antibody (Dako Corporation, CA) at 1∶1000 for 1 h at room temperature.

### Microarray Labelling and Analysis

105 ng of total RNA harvested at 24 h post transfection from the knockdown and overexpression experiments was labelled using the MessageAmp™ Premier amplification according to manufacturer’s instructions (Ambion, NZ). This process involved a first and second strand synthesis, IVT labelling, and purification of the aRNA yield. Subsequently, 8.5 µg of labelled aRNA was hybridised to Affymetrix U133 Plus 2 microarrays. Hybridisation, washing and scanning of the microarrays were performed by the Centre of Proteomics and Genomics (University of Auckland, NZ) according to manufacturer’s instructions.

Bioinformatic analysis was carried out in the ‘R’ statistical environment. The.cel files from each genechip passed quality control using the ‘AffyQCReport’ package in R [28], and were subsequently normalised using the RMA algorithm with background correction [29]. Statistical analysis of different abundance between control (empty vector) and treated cells (wildtype or mutant *FOXL2* overexpression, or mutant *FOXL2* knockdown) was performed on log_2_-transformed data using the LIMMA method in R [30], to generate lists of differentially regulated genes for further functional analysis. Relationships between differentially regulated genes were explored further using the tools GATHER (Gene Annotation Tool to Help Explain Relationships) [31], GeneSetDB (http://genesetdb.auckland.ac.nz/haeremai.html, NZ) [32] and Ingenuity Pathway Analysis software (http://www.ingenuity.com, Redwood, CA).

## Results

### Microarray Evaluation

To identify transcriptional targets of mutant *FOXL2*, we have undertaken two complementary transcriptomic approaches. First, we used expression vectors containing the coding sequence of wildtype or mutant *FOXL2* for overexpression in the mutation negative GCT cell line, COV434. Second, we reduced the expression of mutant *FOXL2* in the GCT cell line heterozygous for the mutation, using siRNA.

**Figure 1:**
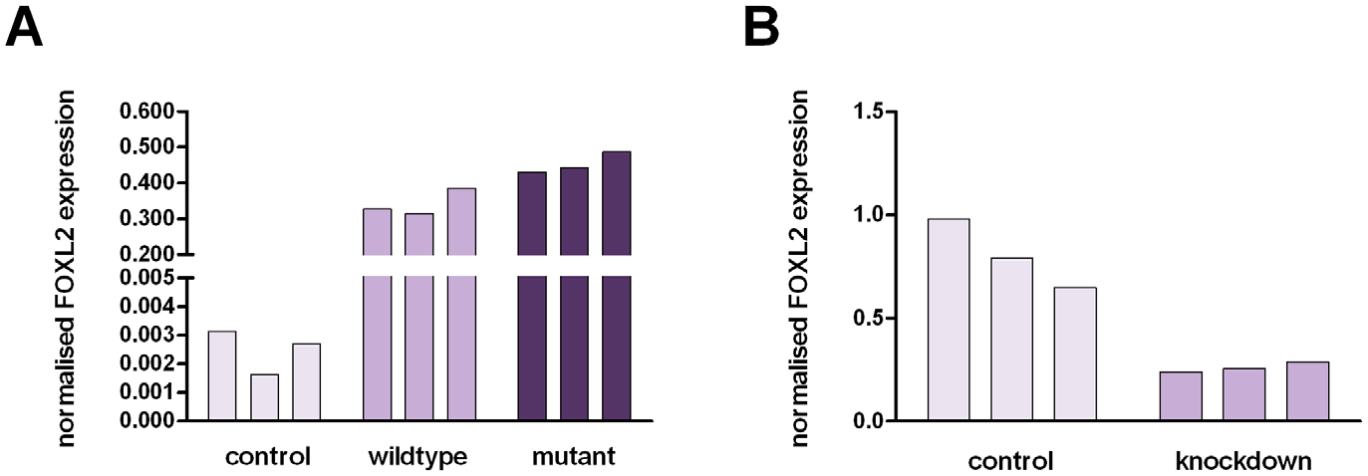
Confirmation of FOXL2 overexpression (A) and knockdown (B) assessed with RT-qPCR. Expression values are plotted as normalised values for each control and treated sample. Errors represent technical error associated with RT-qPCR for each sample. The overexpression of wildtype and mutant FOXL2 resulted in greater than a 120 fold increase in FOXL2 expression in COV434, when compared to control cells. siRNA-targetted cells showed 0.3 times the level of FOXL2 expression observed in control cells following mutant FOXL2 knockdown in KGN.

RT-qPCR was used to confirm the overexpression of wildtype and mutant *FOXL2* in COV434, as well as the knockdown of mutant *FOXL2* in KGN. Figure 1 depicts the changes in *FOXL2* expression assessed using RT-qPCR with the data plotted as normalised expression values seen in triplicate control and treated samples. Triplicate samples were produced by performing three separate transfections on the same occasion. The RT-qPCR results show after overexpression (Figure 1A) and knockdown (Figure 1B), *FOXL2* levels were greater than 120 times, and approximately 0.3 times, the levels observed in the control cells, respectively.

**Figure 2:**
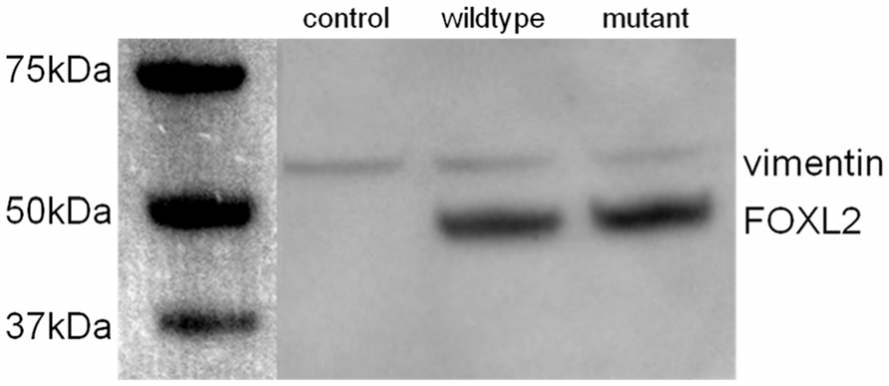
Western blot confirming FOXL2 overexpression in COV434. A clear 45 kDa band in seen in lysates from wildtype and mutant FOXL2 overexpression. This band is absent in control lysates, and similar staining of vimentin across all three lysates confirms FOXL2 overexpression.

In addition, Western blotting was used to confirm the overexpression of wildtype and mutant *FOXL2* expression in COV434 cells (Figure 2). A distinct band is seen in both overexpression lysates at approximately 45 kDa corresponding to the *FOXL2* protein. No such band is observed in the control lysate despite the presence of similarly dense vimentin bands across all samples.

**Figure 3:**
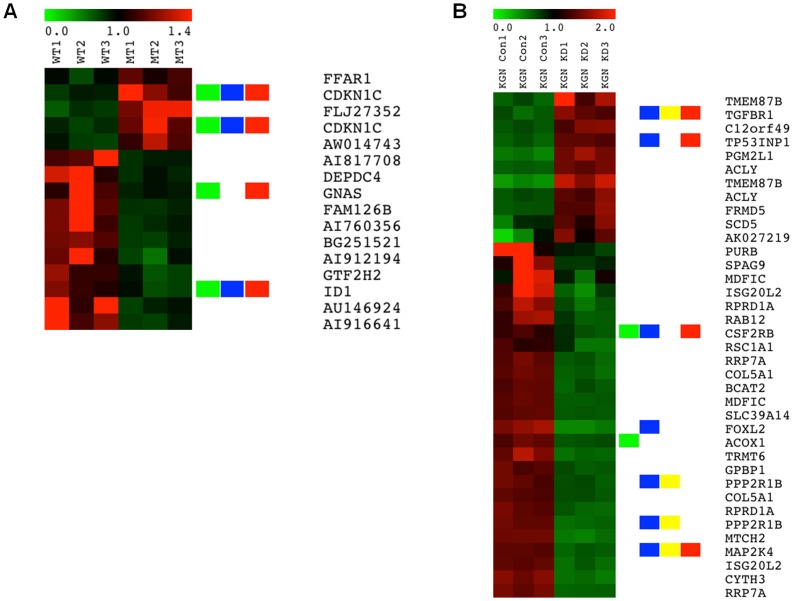
Gene expression profiles associated with mutant FOXL2 overexpression in COV434 cells (A) and mutant FOXL2 knockdown in KGN cells (B). Each heatmap shows signature genes of KGN with LIMMA p<0.01 and absolute fold change >2 and COV434 with LIMMA p<0.01 and absolute fold change <1.4. In the image red refers to upregulation and green is downregulation. The green, blue, yellow and red bars highlight genes annotated by tumourigenesis, cell death, TGF-β signalling and proliferation, respectively.

Having confirmed *FOXL2* overexpression and knockdown in COV434 and KGN cells, respectively, we analysed the gene expression changes induced by altering *FOXL2* expression using Affymetrix microarray gene expression analysis. The microarray results showed a mean *FOXL2* expression log_2_ ratio of −1.77 between control cells and siRNA-targeted KGN cells, which implies a mean 3.41 fold decrease in *FOXL2* expression. No microarray signals were observed for *FOXL2* in either control or transfected COV434 cells. This is expected, given that *FOXL2* expression is absent in this cell line, and that the probeset responsible for detecting *FOXL2* expression is located in the 3′UTR of the gene, which lies outside both the wildtype and mutant *FOXL2* sequences cloned into the overexpression construct. Figures 3A and B are gene expression profiles giving a snapshot of the genes that were shown to be most significantly differentially regulated following overexpression of mutant FOXL2 compared to wildtype FOXL2 in COV434 cells (A) and following mutant FOXL2 knockdown in KGN cells (B). Among these gene lists, we have highlighted genes annotated for functions of tumourigenesis, cell death, TGF-β signalling and proliferation.

### Mutant *FOXL2* Affects the Expression of Direct Targets of Wildtype *FOXL2*

**Figure 4:**
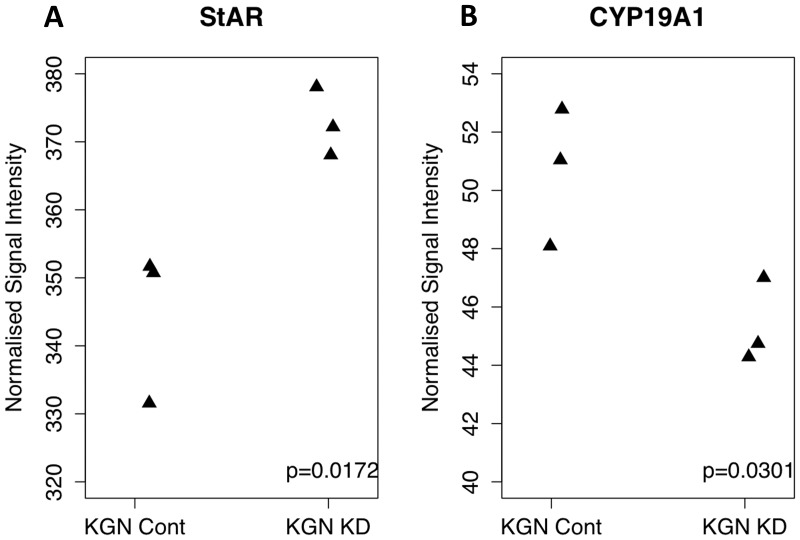
Signal intensity plots from knockdown data for StAR (A) and CYP19A (B). StAR, usually repressed by FOXL2 shows a significant increase (p = 0.01) in expression following the knockdown of mutant FOXL2. CYP19A, usually activated by FOXL2 shows a significant decrease (p = 0.03) in expression following the knockdown of mutant FOXL2. Signal intensities are plotted as RMA normalised data for each genechip. This data leads us to believe that mutant FOXL2 is able to regulate the expression of these FOXL2 targets.

It is unclear whether the 402C>G mutation in the DNA binding domain of *FOXL2* prevents *FOXL2* from altering the expression of its gene targets by altering its binding capacity. To address this issue, we studied the effect of mutant *FOXL2* knockdown on the abundance of the known direct *FOXL2* targets, *StAR* and aromatase. Our hypothesis was that there would be a change in the expression of these RNAs in KGN cells after knockdown of mutant *FOXL2*, suggesting that mutant *FOXL2* was still able to transactivate these targets. Wildtype *FOXL2* normally represses *StAR* gene expression and upregulates the expression of aromatase (*CYP19A1*). Figure 4A shows in our knockdown data, there is a significant increase in the signals from *StAR* following *FOXL2* knockdown in KGN cells (p = 0.01). Similarly, Figure 4B shows a significant decrease in the signals from the aromatase gene between the control and knockdown KGN cells (p<0.03). Together this data suggests that mutant *FOXL2* is able to regulate the expression of these genes, and in its absence, the expression of these genes is altered accordingly.

In addition to looking at the expression of *StAR* and aromatase, we also screened other potential direct FOXL2 targets described by Batista et al (2007), who overexpressed wildtype *FOXL2* in KGN cells. In our knockdown data, the absence of mutant *FOXL2* caused an increase or decrease in the expression of genes shown to be upregulated or downregulated by Batista and colleagues, respectively, by wildtype *FOXL2* (File S1). Such genes that showed consistency between our knockdown and overexpression datasets, and Batista’s gene list, included *SMAD6*, *SOX9*, *SOX4* and *ATF3*. This would be expected if mutant *FOXL2* was still able to regulate these *FOXL2* targets.

**Figure 5:**
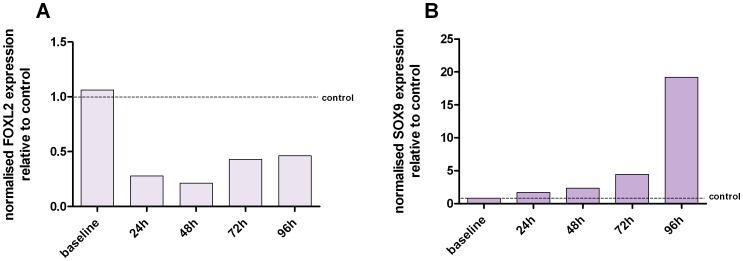
The expression of SOX9 post FOXL2 knockdown in KGN cells. Figure 5A confirms the knockdown of FOXL2 expression and Figure 5B depicts SOX9 expression at each corresponding time point. Each expression value has been normalised to the expression values of three reference genes and have been plotted relative to the control cells (data not shown). Over a 96 h time period, the knockdown in FOXL2 expression resulted in a steady increase in SOX9 expression. Baseline refers to expression levels ascertained before knockdown.

The relationship between *FOXL2* and *SOX9* is a well documented one (Garcia-Ortiz *et al*, 2009; Veitia, 2010). *FOXL2* expression is important in maintaining female sex-gonads, and in its absence, a de-repression of male specific genes occurs. One of these male specific genes is *SOX9*. We used our microarray data to see whether this relationship was evident in the KGN cells following the knockdown of *FOXL2*. The microarray results showed a mean *SOX9* expression log_2_ ratio of 0.59 between control cells and siRNA-targeted KGN cells, which implies a mean 1.55 fold increase in *SOX9* expression. Given this result, we then used RT-qPCR to monitor the changes in the expression of *SOX9* post *FOXL2* knockdown in KGN cells over a greater time period (Figure 5). Indeed with *FOXL2* levels low, the expression of *SOX9* increases steadily over a 96 h time period. This inverse relationship has previously been observed in literature [33], [34], [35], [36], further adding as a form of validation for our knockdown data.

### Cell Line Specific Expression of *FOXL2* Target Genes

**Figure 6:**
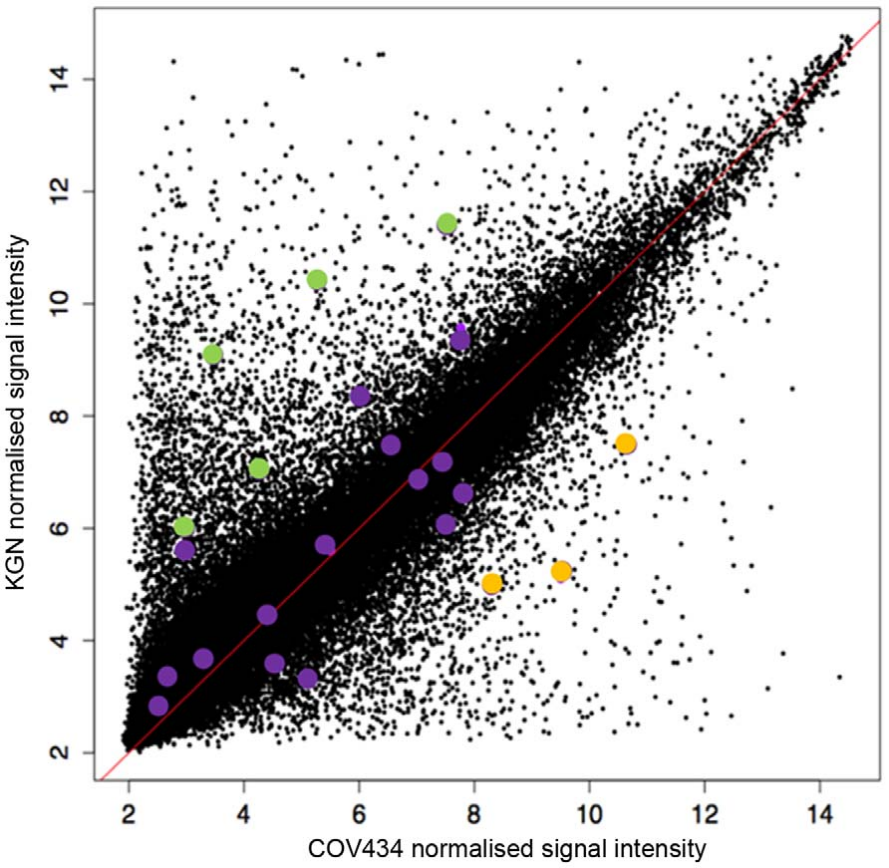
Cell line specific expression of FOXL2 targets. Each point represents a single probeset. Signal intensities have been calculated for each probeset by averaging the signal intensities from the three control samples from the COV434 and KGN array data. Coloured points (purple, green and yellow) represent FOXL2 targets described by Batista et al (2007). Although most genes appear to have similar expression in both cell lines, some genes appear to have preferential expression for either cell line. FOXL2 target genes that are more abundant in KGN include SOX9, SOX4, CYP19A, StAR and FST (as shown by green points). FOXL2 target genes that are more abundant in COV434 include NR5A2, NR4A3 and CDKN2A (as shown by yellow points).

Given that the two GCT cell lines KGN and COV434 differ in their expression of *FOXL2*, we used our microarray data to investigate whether these cell lines also have differing expression in *FOXL2* target genes. To do this, we took the mean signal intensity of each probeset across the KGN control data, and separately across the COV434 control data, and then plotted the relationship between the control data from the two cells lines (as shown in Figure 6). The points in colour represent probesets belonging to possible *FOXL2* targets (outlined by Batista et al (2007) gene list). Overall Figure 6 shows the signal intensities for each probeset are mainly similar between the two cell lines. However, some genes appear to be clearly abundant in one cell line, and nearly absent in the other. This expression pattern is seen among some *FOXL2* targets (purple green and yellow points, as shown in Figure 6). *FOXL2* target genes that are much higher expressed in KGN than COV434 are *SOX9*, *SOX4*, *FST*, *StAR* and *CYP19A1* are the green points. *FOXL2* target genes that are more abundant in COV434 are the two nuclear receptors *NR4A3* and *NR5A2*, and *CDKN2A* are the yellow points.

### Mutant *FOXL2* Regulates Genes Enriched for Functions of Tumourigenesis, Cell Death and Cell Proliferation, in Addition to *TGF-β* Signalling

The overexpression of wildtype and mutant *FOXL2* in COV434 resulted in expression levels within 20% of each as measured by RT-qPCR (refer to Figure 1). Therefore, in order to understand the pathogenic effect of mutant *FOXL2*, we compared the transcriptomes of COV434 cells overexpressing wildtype and mutant *FOXL2*. LIMMA was used as a ranking tool and identified 340 annotated genes (p<0.01, fold changes ranging from −1.69 to 1.83) that were differentially regulated between the two treatments, that is, genes that are regulated by mutant *FOXL2*.

We then used the software Ingenuity Pathway Analysis (IPA) to identify relationships, functions and pathways of relevance over-represented in the list of genes differentially expressed between wildtype *FOXL2* and mutant *FOXL2* transfected COV434 cells. The IPA analysis revealed that the differentially expressed genes were enriched for functional annotations of tumourigenesis (p = 1.56E-5), cell death (p = 2.70E-7), and cell proliferation (p = 6.66E-7). Many of the genes were shown to belong to all three categories including *SMAD3*, *BMPRB1*, *CDKN1A*, *CDKN2A*, *CDK6*, *BTG*, *JUN* and *INHBA* (File S2).

**Figure 7:**
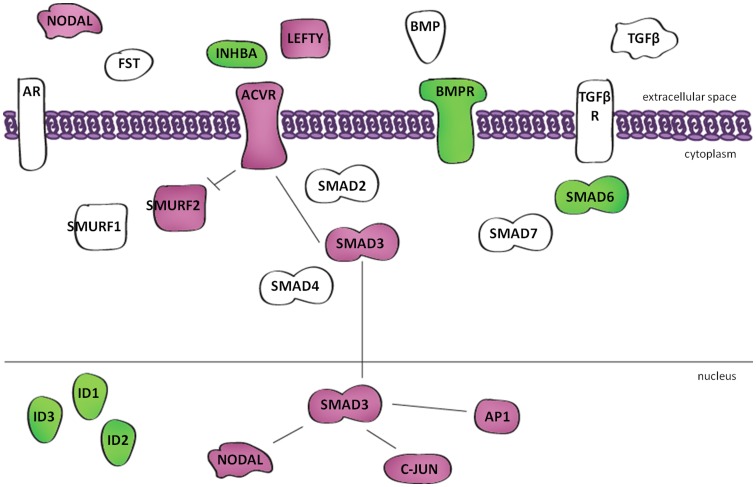
Mutant FOXL2 regulates the expression of genes in the TGF-β signalling pathway. Figure schematically highlights those genes in the TGF-β pathway that are regulated by mutant FOXL2. All genes in magenta show increase in expression, genes in green show decrease in expression.

In addition, the IPA analysis revealed that many of the genes (refer to Figure 7) shown to be regulated by mutant *FOXL2* mapped to the *TGF-β* pathway, including *SMAD* family members *3* and *6*, the signalling ligand *INHBA*, and receptors belonging to *BMP* and activin. *TGF-β* signalling was also found to be enriched when using the gene annotation tools GATHER (path:hsa04350, p<0.0001, Bayes Factor 7) and GeneSetDB (p<0.001, FDR 0.19). To determine whether the relationship between mutant *FOXL2* target genes and *TGF-β* signalling was significant, we performed a robust permutation analysis comparing the enrichment of *TGF-β* signalling genes in our gene list, to the enrichment of *TGF-β* signalling genes in 10,000 lists of randomly generated genes of equal size to our gene list (File S3). In this figure, the blue dotted lines represent the 5^th^ and 95^th^ percentile, respectively. The green arrow indicates that our data lies above the 95^th^ percentile of the randomly generated data lists, indicating there is a <5% probability that the genes are enriched for *TGF-β* signalling by chance alone. Interestingly, *TGF-β* signalling was also significantly enriched when studying gene lists generated by comparing KGN cells with *FOXL2* knockdown relative to the control, and also COV434 cells overexpressing mutant *FOXL2* compared to the control (data not shown). However this enrichment was not seen when comparing wildtype *FOXL2* overexpressed COV434 cells to control cells. Furthermore an opposite expression pattern was seen when comparing gene lists from mutant knockdown KGN cells and mutant overexpression COV434 cells in these *TGF-β* genes including *SMAD3* and *INHBA*, further highlighting the involvement of the mutant gene in *TGF-β* signalling.

## Discussion

The 402C>G mutation in *FOXL2* described by Shah et al has been observed in nearly all cases of adult type GCTs that have been investigated worldwide. It is likely this mutation is pivotal in the pathogenesis of GCTs, as the *FOXL2* gene is important in normal granulosa cell function. No other tumour type identified to date has the same *FOXL2* mutation and it seems striking that it occurs in the same position of the gene in GCT, yet little is known about its pathogenic mechanism of action. We have adopted a transcriptomic approach to study the effect of the *FOXL2* mutation by analysing how gene expression in GCT cell lines is altered after perturbing the expression of either wildtype or mutant *FOXL2*. The aim of this study was to identify transcriptional targets of the mutant, and thereby identify aspects of its pathogenicity.

The 402C>G mutation resides in the DNA binding domain of *FOXL2* and results in an amino acid change from a cysteine to a tryptophan (C134W). However, computer modelling suggests that this amino acid substitution does not alter the conformation of the domain, suggesting the mutant protein can still bind DNA [12]. One possible explanation to describe mutant *FOXL2*’s behaviour, is that although able to bind DNA, the mutant is unable to bind to targets usually directly regulated by wildtype *FOXL2*. This would mean that the mutant *FOXL2* is unable to recognise and bind the *FOXL2* response element in direct wildtype *FOXL2* gene targets. However, the work of Benayoun et al (2010) used reporter constructs to demonstrate the transactivation capability of mutant *FOXL2* on known wildtype *FOXL2* targets. With the exception of one promoter belonging to *GRAS* (GnRH receptor activating sequence), in which the mutant appeared to be hyperactive, the mutant behaved similarly to the wildtype protein on all other accounts [18]. We have used our microarray data to further investigate the idea that mutant *FOXL2* regulates the expression of a different suite of genes compared to wildtype *FOXL2*.

We studied the changes in expression of known direct *FOXL2* targets in our KGN knockdown data to see if mutant, compared to wildtype *FOXL2,* was able to differentially regulate the expression of known *FOXL2* targets. Many targets of FOXL2 have been individually identified such as *GnRHR, alpha-GSU, FST, FSH-beta and CYP17A1* as well as many targets obtained in several transcriptomic studies and genome-wide ChIP-on-chip experiments [33], [35], [37], [38], [39]. However we chose to focus our analysis on two transcriptional targets that are directly relevant in granulosa cell biology, namely *StAR* and the gene encoding aromatase *(CYP19A1)* [38], [40], [41]. *StAR* is a marker of late differentiation of granulosa cells in pre-ovulatory follicles and catalyses the translocation of cholesterol from the outer to the inner mitochondrial membrane, where it can be subsequently processed to yield steroid hormones. Also involved in steroidogenesis, aromatase is the enzyme responsible for the conversion of androgens to oestrogens in granulosa cells. We compared the normalised signal intensities of *StAR* and aromatase across our knockdown data in KGN cells and revealed that mutant *FOXL2* was still able to alter the expression of these *FOXL2* targets. Wildtype *FOXL2* normally represses *StAR* gene expression [42] and upregulates the expression of aromatase, as consistent with our understanding of ovarian biology. In our *FOXL2* knockdown data, we saw a significant increase in *StAR* expression (p = 0.01) and decrease in aromatase expression (p = 0.03). As we are likely to be silencing both wildtype and mutant *FOXL2* alleles in the KGN line, we are suggesting that a reduction in the combined amount of *FOXL2* leads to an altered regulation of the expression of these genes. However, given that we are unable to separate loss of wildtype from mutation *FOXL2* function in this heterozygous cell line, we can not discount the possibility that mutant *FOXL2* is unable to bind to *StAR* and aromatase promoters, and therefore we might not be able to measure this change in expression.

Unfortunately, in our wildtype and mutant overexpression data, the low level of expression of some *FOXL2* target genes made it difficult to assess the effect of *FOXL2* overexpression in COV434 cells. In this case, a more effective method of investigating mutant *FOXL2*s binding on wildtype *FOXL2* targets may be to see if there was an enrichment of genes containing the *FOXL2* response element in wildtype and mutant overexpressed COV434 cells compared to control cells. However, given the lack of specificity and discrepancy between the published *FOXL2* response elements [21], [40], [43], [44], we chose to assess mutant *FOXL2* binding indirectly through looking at changes in gene expression.

In addition to *StAR* and aromatase, we performed a similar screening method for other potential *FOXL2* targets that were originally identified by Batista et al (2007), who overexpressed wildtype *FOXL2* in KGN cells [38]. We looked to see whether there were consistent changes in gene expression when we altered the levels of wildtype and mutant *FOXL2*. In our knockdown data in KGN, the absence of mutant *FOXL2* caused a significant increase or decrease in the expression of few of the genes that Batista et al (2007) showed to be downregulated or upregulated, respectively, by wildtype *FOXL2* (p<0.05). This would be expected if mutant *FOXL2* was still able to regulate these wildtype *FOXL2* targets. File S1 details a full list of genes that showed consistency with the Batista et al (2007) gene list across both our knockdown and overexpression datasets. When comparing Batista et al’s gene list with our knockdown data, only 5% of the gene list showed consistency in direction of fold change, that is showing opposite expression patterns that would be expected when comparing gene knockdown and gene overexpression. Similarly when comparing our wildtype and mutant overexpression data with Batista et al (2007), we see only 2% of their gene list showing consistency with ours.

Such direct comparisons between gene lists can be difficult due to the differences in methodologies between the two groups. For example, Batista et al (2007) performed their experiments in KGN using a double transfection protocol, whereas we performed a single transfection protocol in both cell lines. Additionally, considering the dynamic nature of transcriptome responses, the differences in the timepoints for analysis (their 48 h post transfection versus our 24 h post transfection) may also affect the similarities, or lack thereof. As we have shown in Figure 6, the two cell lines possess differences in their global gene expression profiles, ultimately making comparisons between the two experiments problematic. Although our knockdown data and Batista’s overexpression data was performed in KGN, we are likely to be altering both mutant and wildtype alleles, where as Batista et al have only increased expression of the wildtype allele, which can also explain the differences between our gene lists. Furthermore, the expression data from each experiment was obtained from two different array platforms, different types of statistical analyses were performed to produce a working gene list and the known issue of batch effects in microarray meta-analysis could also induce spurious differences in gene activation [45]. Finally, though paradoxical, the simple comparison of knockdown to overexpression, does not always yield consistent results even when assessed completely in parallel [46]. Indeed some of these caveats discussed may also explain the lack of overlap in all of the *FOXL2* transcriptome studies and between the COV434 and KGN arrays herein, yet it was reassuring to see overlap does occur among some biologically relevant genes. One gene of importance that did show such concordance was *SOX9*.

*SOX9* is the downstream effector of the SRY gene, the Y-linked signal responsible for testis determination in male [47]. Recently, studies have shown that the ovary is constantly suppressing the expression of male-specific genes throughout life [36]. *FOXL2* expression is important in maintaining the female sex gonads, even in adulthood, and in its absence a de-repression of male-specific genes such as *SOX9* occurs [35]. Ulenhaut et al showed in the absence of *FOXL2*, the follicular structure of the ovary began to take on the structure of seminiferous tubules, and granulosa cells are reprogrammed into their male counterparts Sertoli cells. As our *FOXL2* knockdown data showed a significant increase in *SOX9* expression, we were interested to see the long-term effects of reduced *FOXL2* expression on the expression of *SOX9*. As Figure 5 depicts, *SOX9* expression continued to steadily rise in the absence of *FOXL2* over 96 h following *FOXL2* knockdown in KGN. Therefore, it was appropriate for us to undertake a microarray analysis of gene expression at a 24 h timepoint when *SOX9* levels are lowest following *FOXL2* knockdown. Given that we saw no other male specific genes, such as *DMRT1* being significantly upregulated in our knockdown data, we are confident that the KGN granulosa cells have yet to begin their transition to Sertoli-like cells.

Given that the two GCT cell lines KGN and COV434 differ in their expression of *FOXL2*, we used our microarray data to investigate whether these cell lines also have differing expression in *FOXL2* target genes. Figure 6 depicts the global differences in gene expression between the two cell lines, with the *FOXL2* targets outlined by Batista et al (2007) highlighted in purple. Interestingly *SOX9* is the top gene shown in Figure 6 to have a significantly more abundant expression in KGN cells as opposed to COV434, indicating there must be other mechanisms at play keeping *SOX9* expression under control in the *FOXL2* lacking cell line COV434. Other genes that are more abundantly expressed in KGN, than COV434 include the two oestrogen synthesis genes *StAR* and aromatase, however COV434 cells, as well as juvenile-type GCTs are both capable of oestrogen production [23], [48] and there has yet to be any data published showing oestrogen production to be higher in adult-type GCTs than juvenile-type. It appears only three *FOXL2* target genes have significantly higher abundance in COV434 than KGN cells, these genes being the two steroidogenic receptors *NR5A2* and *NR4A3* (also known as *SF-2* and *NOR1* respectively), and *CDKN2A*. Given the preferential expression of *FOXL2* target genes in either KGN or COV434 cell lines, this might suggest to us regulation of these targets may be not always be directly under *FOXL2* control, and perhaps alternative mechanisms may be in place. However, it is possible the differences in the expression of *FOXL2* targets is partially accounted for by the stage of granulosa cell maturation and the different genetic backgrounds of the two individuals from which the cells lines were derived, the differences in type of ‘control’ used (empty plasmid versus non-targeting siRNA), and lastly, the likelihood that both cell lines have undergone many other genomic hits after the perturbation of the *FOXL2* locus. Although we are not able to use our data to provide explanations to account for the differences in *FOXL2* target gene expression between COV434 and KGN, what we have done is confirm at a transcriptomic level what is already known at a pathological level; that adult and juvenile-type GCTs are quite different diseases that have arisen from the same cell type, but from likely two different mechanisms.

To gain further insights about genes regulated by mutant *FOXL2*, we used the software IPA, a database system for understanding how proteins work together to effect cellular change. The IPA analysis revealed that the genes shown to be regulated by mutant *FOXL2* (gene list created from comparing the transcriptome of COV434 cells overexpressing wildtype or mutant *FOXL2*) were enriched for functional annotations of cell death, cell proliferation and tumourigenesis. It has been previously shown that *FOXL2* activation tends to promote cell cycle arrest at the G1/S checkpoint through direct regulation of cyclin dependant kinases and their inhibitors [37]. It appears that mutant *FOXL2*, like wildtype *FOXL2*, upregulates the expression of the well known tumour suppressor *CDKN1C (p57)*, in addition to *CDKN1A (p21/WAF).* Similarly, mutant *FOXL2* caused a decrease in the expression of *CDKN2A* (p16/INK4a), which was shown by Arcellana et al to have reduced expression in 58% of adult GCTs [49]. So although it may appear overall that the changes in gene expression by mutant *FOXL2* would be protective in the development of GCTs, it is likely that the differences in expression of a limited number of key genes are able to tip this balance in favour of tumourigenesis.

In addition, the IPA analysis revealed that many of the genes shown to be to be regulated by mutant *FOXL2* mapped to the *TGF-β* signalling pathway. It is not surprising that this signalling pathway was enriched, as it has been previously suggested that it is likely that mutant *FOXL2* has altered interactions with SMAD transcription factors and effects of the TGFβ and BMP family signalling [19]. Although Figure 7 only depicts those genes traditionally seen in *TGF-β* signalling diagrams, our gene list also showed the movement of other SMAD gene targets including *CDKN1A, DLX3 and GADD45B*. It is reassuring that we identified this pathway from our non-hypothesis driven bioinformatic analysis of transcriptomic data from different cell line studies. The *TGF-β* signalling pathway has been implicated in many human diseases including cancer. *TGF-β* signalling, originally renowned for its anti-proliferative activity, is now considered to demonstrate both tumour suppressor and oncogenic properties [50], [51]. In the current paradigm, the suppressor activities dominate in normal tissue, but during tumourigenesis, changes in *TGF-β* expression and cellular responses tip the balance in favour of its oncogenic activities. This process usually involves a decrease in the expression of the signalling ligand and receptor, decreased SMAD levels or activity, or a compromise in the effector function of the suppressor arm of this pathway [52]. Further supporting this idea, is the loss of expression of the antiproliferative signalling ligand *INHA*, and loss of *BMPR1B* receptor expression, as well as in increase the expression of the oncogene *JUN*.

It is interesting to note that the IPA functional annotations of cell death, cell proliferation, tumourigenesis and *TGF-β* signalling were also shown to be significantly enriched, when studying the gene lists derived from the LIMMA analysis of the mutant *FOXL2* knockdown in KGN and the overexpression of mutant *FOXL2* in COV434. However, when performing the same analysis with a gene list compiled from the overexpression of wildtype *FOXL2* in COV434 compared to control treated cells, none of these annotations were shown to be significant. In fact, very few annotations and pathways were shown to be significantly enriched in this particular analysis. Therefore this finding demonstrates that altering expression levels of wildtype *FOXL2* alone is not important for tumour development, but it is the 402C<G mutation itself in *FOXL2* that is responsible for altering biological functions to contribute to GCT pathogenesis. Furthermore, it also suggests that in our gene knockdowns, it is likely that we are silencing the mutant allele as well as the wildtype allele, as if only the wildtype allele was silenced, the mutant allele expression would remain, and the gene list data would resemble the mutant overexpression data in COV434.

### Conclusions

In conclusion, we have identified aspects of the pathogenicity of mutant *FOXL2* through studying its transcriptional targets after perturbing its expression in two GCT cell lines COV434 and KGN. The overexpression of wildtype and mutant *FOXL2* in COV434, and the silencing of mutant *FOXL2* expression in KGN, has revealed that mutant *FOXL2* is able to differentially regulate the expression of many genes, including two well known *FOXL2* targets *StAR* and CYP19A. In addition, we have confirmed at a transcriptomic level, the significant difference in gene expression between adult and juvenile type GCTs, an important consideration for future therapeutic work. We have shown that many of the genes regulated by mutant *FOXL2* are clustered into functional annotations of cell death, proliferation and tumourigenesis. In addition, these genes are significantly enriched for *TGF-β* signalling, and we suggest that deregulation of this key antiproliferative pathway is perhaps one way mutant *FOXL2* contributes to the pathogenesis of adult-type GCTs.

## Supporting Information

File S1 Genes shown to have similar regulation when comparing our gene list with that generated by Batista et al (2007).
(XLSX)

File S2 Outline of genes regulated by mutant FOXL2 shown to be enriched for functions of tumourigenesis, cell death and proliferation.
(XLSX)

File S3 Permutation analysis to test for enrichment of TGF-β signalling in our data. In this figure, the dotted blue lines represent the 5^th^ and 95^th^ percentile respectively. The green arrow indicates our data lies above the 95^th^ percentile of randomly generated lists, thus roving the enrichment for TGF-β signalling seen in our gene lists is significant.
(TIF)
