# Supplementary figures and images for: The Transcriptional Targets of Mutant FOXL2 in Granulosa Cell Tumours
Source: PLoS One. 2012 Sep 28;7(9):e46270. doi: 10.1371/journal.pone.0046270 (PMC3460904; doi:10.1371/journal.pone.0046270)

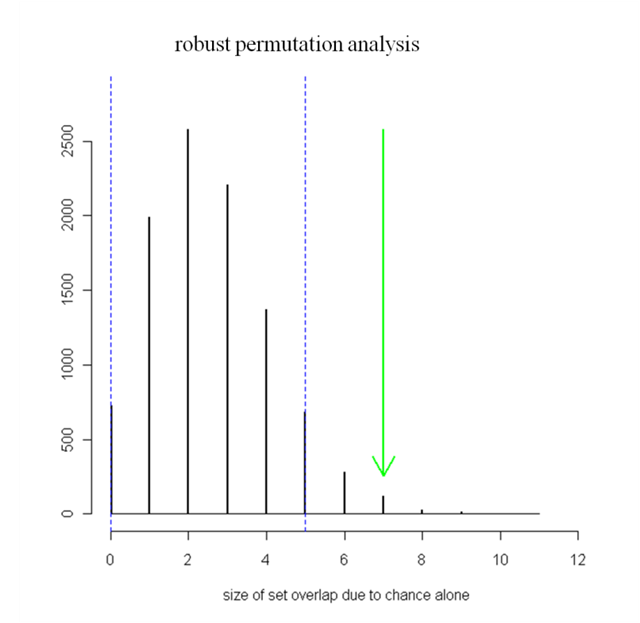

Supplement: File S3 — Permutation analysis to test for enrichment of TGF-β signalling in our data. In this figure, the dotted blue lines represent the 5th and 95th percentile respectively. The green arrow indicates our data lies above the 95th percentile of randomly generated lists, thus roving the enrichment for TGF-β signalling seen in our gene lists is significant. (TIF) [file pone.0046270.s003.tif]
